# Requirement to change of functional brain network across the lifespan

**DOI:** 10.1371/journal.pone.0260091

**Published:** 2021-11-18

**Authors:** Majid Saberi, Reza Khosrowabadi, Ali Khatibi, Bratislav Misic, Gholamreza Jafari

**Affiliations:** 1 Institute for Cognitive and Brain Sciences, Shahid Beheshti University, G.C. Tehran, Iran; 2 Centre of Precision Rehabilitation for Spinal Pain (CPR Spine), School of Sport, Exercise and Rehabilitation Sciences, University of Birmingham, Birmingham, United Kingdom; 3 McConnell Brain Imaging Centre, Montréal Neurological Institute, McGill University, Montréal, QC, Canada; 4 Physics Department, Shahid Beheshti University, Tehran, Iran; 5 Institute of Information Technology and Data Science, Irkutsk National Research Technical University, Irkutsk, Russia; Hunan Normal University, CHINA

## Abstract

Many studies have focused on neural changes and neuroplasticity, while the signaling demand for neural modification needs to be explored. In this study, we traced this issue in the organization of brain functional links where the conflictual arrangement of signed links makes a request to change. We introduced the number of frustrations (unsatisfied closed triadic interactions) as a measure for assessing "requirement to change" of functional brain network. We revealed that the requirement to change of the resting-state network has a u-shape functionality over the lifespan with a minimum in early adulthood, and it’s correlated with the presence of negative links. Also, we discovered that brain negative subnetwork has a special topology with a log-normal degree distribution in all stages, however, its global measures are altered by adulthood. Our results highlight the study of collective behavior of functional negative links as the source of the brain’s between-regions conflicts and we propose exploring the attribute of the requirement to change besides other neural change factors.

## 1. Introduction

The brain is a malleable organ that adapts to new situations. These situations may have internal or external sources; internally such as genetic polymorphisms, recovery of neural injuries, neural development, systematic reorganization, and externally like behavioral stimuli, social experiences, cognitive-enhancing activities [1–7]. The alteration also ranges from synaptic level to large-scale networks and may be related to the structure or function of the nervous system [8]. In recent years, state-of-the-art neuroimaging techniques facilitated tracing structural reformation and physiological adaptation of the human brain [9].

The ability of the neural change is called neuroplasticity [8, 10]. Certainly, strong neuroplasticity helps to modify the neural system in a better way. It also entitles structural neuroplasticity when it involves the neural formation and connection and functional neuroplasticity in case of functional modification [8]. Also, at the network level, functional neuroplasticity pursues the policy of changing regional interactions to improve brain efficiency [11].

Now, an important question has arisen that neuroplasticity should respond to which requirement? How much is the amount of the changing demand? Less attention has been paid to these questions yet although this may be an essential issue. For instance, suppose an isolated infant with low exposure to environmental and social experiences, although developmental factors enhance the ability of neural changing, isolation prevents providing a situation that neural system require to change, so desired neural changes could not occur and the infant loses the chance of normal development. Accordingly, we claim that changing demand is an influential attribute in neural change studies and needs more attention.

One of the cases is the study of the requirement to change at the network level where network plasticity is a popular concept [12, 13] and lots of researches are devoted to brain network alteration [14–16]. If the issue is related to the organization of functional links, we can utilize standard techniques of complex networks. In this regard, we decided to study the requirement to change of functional brain network using structural balance theory [17, 18]. Also, we studied this issue across the lifespan to obtain a better view of the approach since there is proper knowledge on the quality of neural change over the lifespan [16, 19, 20] and we can interpret neuroplasticity and requirement to change simultaneously.

**Fig 1:**
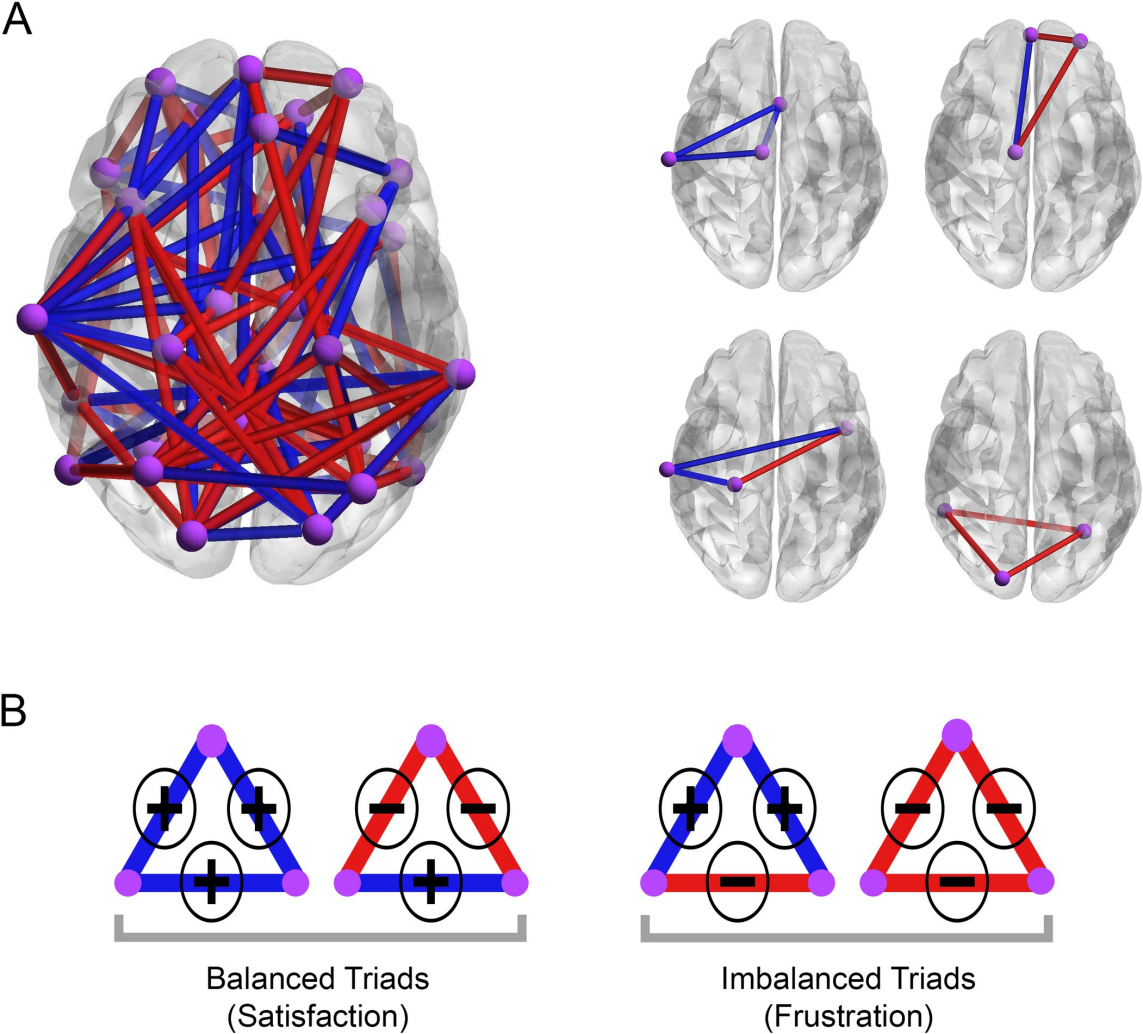
**(A)** A brain signed network and formation of closed triadic relations. Violet nodes reflect brain regions, and blue and red links correspond to positive and negative functional connections. **(B)** Classification of possible closed triadic types based on inter-entity conflicts and Heider’s balance theory.

What is the structural balance theory? The theory explains dissonance in a signed network by exploring the triadic associations between connections [18]. It originally refers to Fritz Heider’s studies on attitude change [17] then was described in the context of friends and foes [21]. The theory classifies interpersonal relationships between three familiar persons into balanced and imbalanced triads [22] (Fig 1B). Balanced association: when a friend of a friend or an enemy of an enemy is a friend; imbalanced associations: when a friend of a friend or an enemy of an enemy is an enemy. Based on the theory, entities of an imbalanced triad are frustrated about their aggregation and they endure pressure to change the quality of their mutual relations to make triadic relationship balanced [17, 22]. Following Heider’s researches, Cartwright and Harary extended the theory for signed networks that have numerous mentioned triadic types [18, 23]. In addition, based on the principle of minimum frustration [24], the signed network must resolve its frustrations (imbalance triads) by changing the signed links to bring itself toward a more stable state with lower energy [25, 26]. So we claim that the number of frustrations in a signed network indicates how much the network needs changes.

If we model the brain as a signed network where synchrony and anti-synchrony between regional brain activations determine signed links [27] (Fig 1A), we can expect that the brain signed network tries to resolve its frustrations by changing the sign of links. Therefore, we can regard the number of triadic frustrations presented in the functional signed network as a measure of the requirement to change of functional brain network.

In this work, we decided to compare the number of frustrated triads that are appeared in the resting-state signed networks of healthy subjects to investigate whether the requirement to change of functional brain network alters throughout the lifespan stages. We chose this multi-stage comparison because of the almost well-known trend of neural changes and neuroplasticity over the lifespan. Because of the important role of negative links in network balance and frustration formation, we also tried to investigate topological features of brain negative subnetwork.

## 2. Results

**Fig 2:**
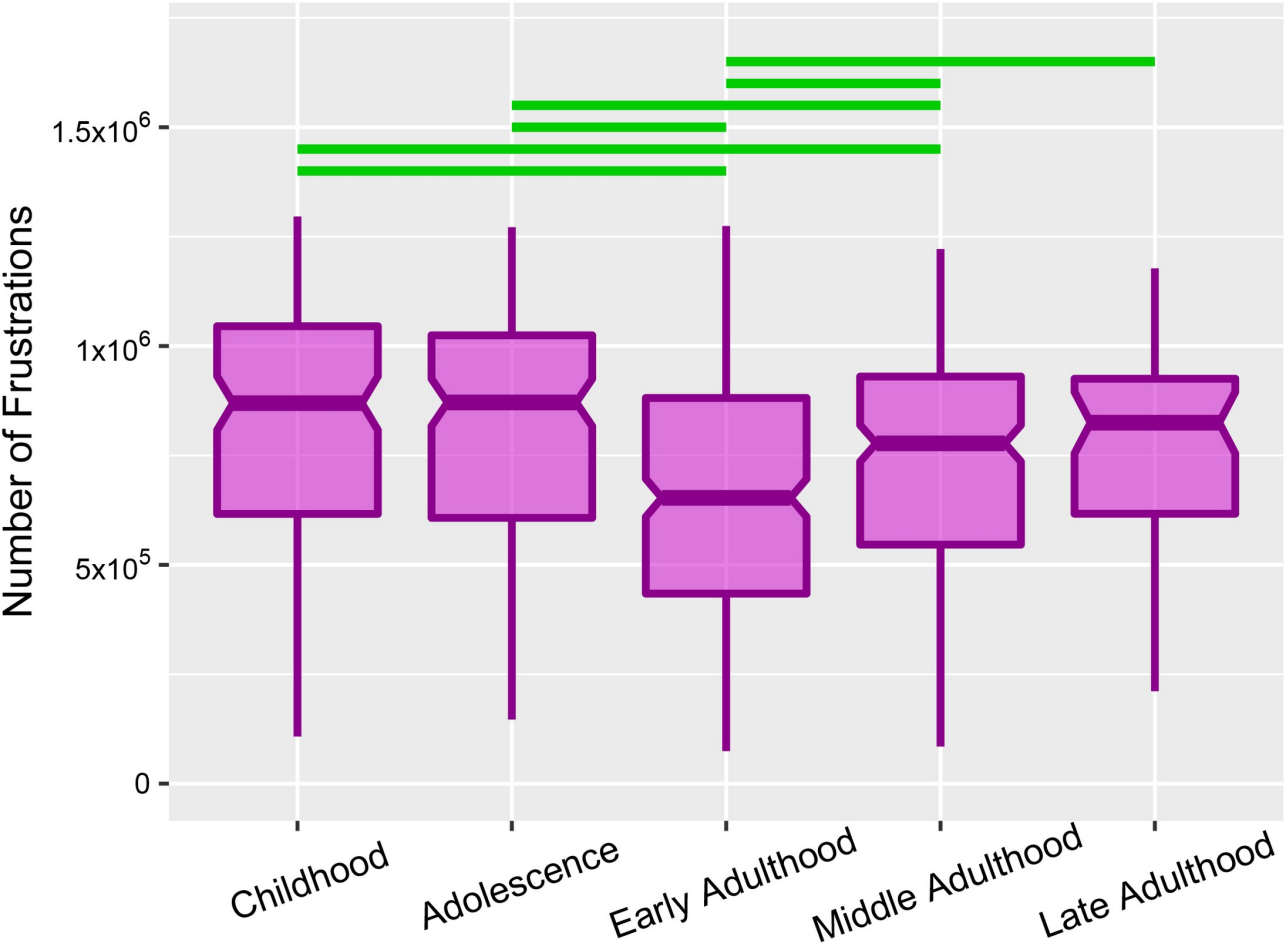
Multiple-group comparisons of frustrated triads at various stages of the lifespan. Horizontal lines of boxes indicate medians and notches determine 95% confidence interval for the medians. Green lines represent significant group differences after multiple comparison corrections (p-value **≤** 0.05).

**Fig 3:**
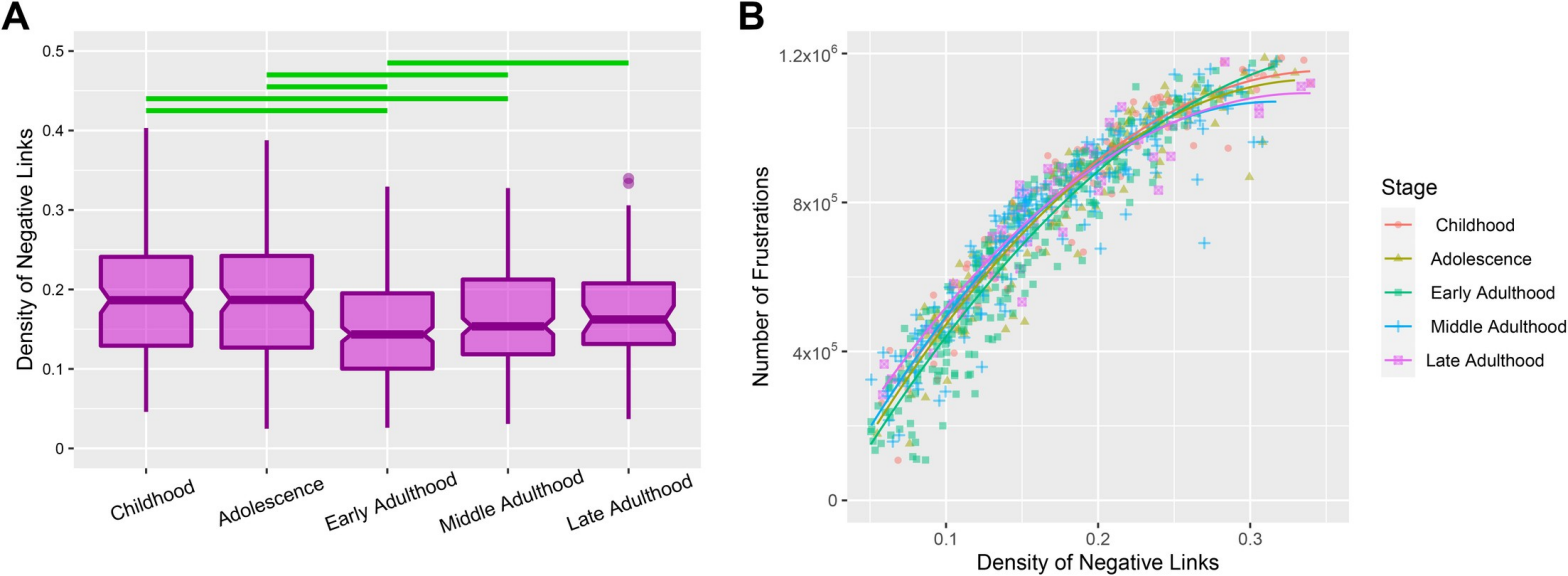
Multiplicity of brain negative connections. **(A)** Alteration of negative link density of resting-state networks throughout lifespan stages. The notched regions of boxes indicate the median and 95% confidence interval of the medians. Horizontal green lines denote significant differences between stages after correction of multiple comparison effect (corrected p-value **≤** 0.05). **(B)** A positive non-linear correlation between the density of negative links and the number of frustrations. Each point represents the density of negative links and the number of frustrations corresponding to a subject. The colored shapes indicate developmental stages. The colors of lines also differentiate quadratic functions fitted to the points of each stage.

**Fig 4:**
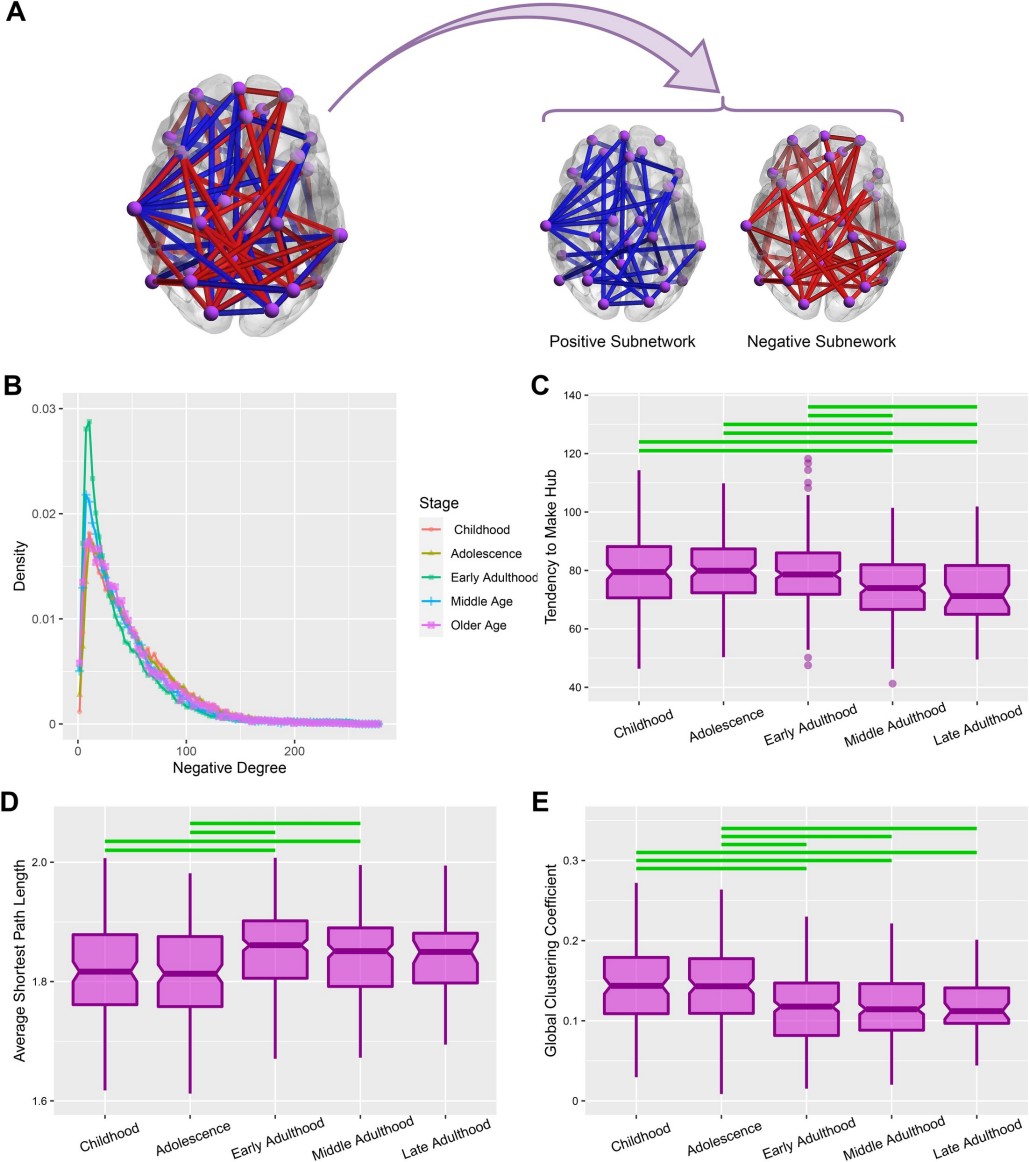
Topological analysis of brain negative subnetwork. **(A)** Splitting brain signed network to positive and negative subnetworks. **(B)** Stagewise degree distributions of brain negative subnetwork. **(C-E)** Alteration of three global features of hubness, efficiency and clustering of brain negative subnetwork over the lifespan stages. These features are measured using Tendency to Make Hub, Average Shortest Path Length, and Global Clustering Coefficient. The notched regions of boxes indicate median and 95% confidence interval of the median, and horizontal lines denote between stages significant differences after multiple comparison correction (corrected p-value **≤** 0.05).

In this study, we wanted to assess the requirement to change of functional brain network using balance theory, then compare it over the lifespan stages and investigate relevant network features. So we decided to divide healthy subjects of two publicly available repositories of ABIDE [28] and Southwest [29] into Erikson’s developmental stages including childhood (age: 6–12 y.o.), adolescence (age: 12–18 y.o.), early adulthood (age: 18–40 y.o.), middle adulthood (age: 40–65 y.o.) and late adulthood (age: greater than 65 y.o.) [30]. Afterward, we preprocessed resting-state functional images of the subjects. Then we took out activity patterns of regions of interest from preprocessed functional images based on Shen’s parcellation [31]. Table 1 represents the demography of the subjects separated by study sites after applying preprocessing criteria. In the following, we computed a functional connectivity from the extracted activity patterns of each subject, and binarized values of connections to obtain signed networks. After providing a sign network for each subject, we counted triadic frustrations (dependent variable) that we observed in each signed network. Finally, we performed a multiple-group comparison (statistical design) between subjects of different lifespan stages (independent variable) in terms of the number of frustrations (Fig 2). Because of the important role of negative links in frustration formation and brain network balance, we also investigated the alteration of topological measures of brain negative subnetworks including network size, degree distribution, efficiency, clustering, and hubness over the lifespan stages (Figs 3 and 4). S1 Fig also shows the described procedure of this study.

**Table 1:** Subject demographics.

Dataset	Study	Child	Adolescent	Early Adult	Middle Adult	Late Adult	Total Subjects
		(6–12)	(12–18)	(18–40)	(40–65)	(65 <)	
Southwest	Southwest University	-	-	173	211	48	432
ABIDEI	NYU Langone Medical Center	28	31	26	-	-	85
	San Diego State University	-	20	-	-	-	20
	University of Michigan	13	37	9	-	-	59
	University of Utah School of Medicine	1	6	25	-	-	32
	Yale Child Study Center	9	15		-	-	24
ABIDEII	ETH Zürich	-	2	14	-	-	16
	Georgetown University	36	12	-	-	-	48
	NYU Langone Medical Center	24	2	1	-	-	27
	San Diego State University	10	12	-	-	-	22
	Trinity Centre for Health Sciences	-	10	5	-	-	15
	University of Utah School of Medicine	-	1	12	-	-	13
**Total Subjects (Female)**		121 (32)	148 (32)	265 (117)	211 (140)	48 (29)	793 (350)
**Age (Mean ± SD)**		9.7 ± 1.4	14.8 ± 1.69	25.04 ± 4.8	54.06 ± 6.53	71.31 ± 3.83	31.31 ± 19.78

### 2.1. Requirement to change

In the beginning, we counted the number of frustrated triads as a measure of the requirement to change of functional brain network. In the following, we tested the normality of the number of frustrations for the subjects of each stage separately. Consequently, the non-normality of the distributions (S2 Fig) directed us to non-parametric statistics. Since we wanted to carry out a stagewise comparison between the calculated numbers of frustrations, we decided to employ the non-parametric Kruskal-Wallis test that is also feasible to our unequal sample sizes. Kruskal-Wallis test revealed a significant difference between the number of frustrations over the lifespan stages (H = 44.51, d.f = 4, p-value = 5.03e-09). We also applied Dunn’s post hoc test to recognize which two stages had significant differences (S1 Table). Corresponding to the statistical analysis, Fig 2 shows a u-shape functionality of the requirement to change over the lifespan where the number of frustrations stands at the highest level in childhood, declines by development, and reaches the lowest level in early adulthood, then grows by aging in late stages.

We also performed the test separately for each frustration type, triads with three negative links and triads with one negative and two positive links. S3 Fig indicates significant differences in both of them. In addition, we set several thresholds on functional connections before signed network formations and carried out the comparison for partially connected networks. S4 Fig demonstrates rejection of the null hypothesis even after thresholding. Also, we performed the comparison on fully-connected functional brain networks obtained from Automated Anatomical Atlas (AAL) parcellation [32] and observed the same alterations and significant differences for all stages except the late stage (S5 Fig).

### 2.2. Negative link abundance

As regards in Fig 1B, frustrated triads have more negative links compared to balanced triads. In other words, the probability of contribution of a negative link in frustration formation differs from satisfaction formation. So we decided to investigate negative link density over the lifespan stages and study the relation between the density of negative links and the number of frustrations in the functional brain network.

To explore alteration of negative link density across the lifespan stages, initially, we tested the normality of negative link density of subjects. Since the negative link density of every stages had a non-normal distribution (p-value of Shapiro-Wilcoxon tests lower than 0.0001), we employed the Kruskal-Wallis test to investigate the dissimilarity between the stages.

The test indicated significant differences between negative links density of lifespan stages (H = 33.82, d.f = 4, p-value = 8.11e-07). Fig 3A shows medians of the stages and denotes significant Dunn’s post hoc comparisons. S2 Table also presents between stage statistics.

Fig 3A shows that negative link density has a trend similar to the number of frustrations throughout the lifespan, so we decided to investigate the relation between negative link density and the number of frustrations. Consequently, we found a non-linear correlation between the two mentioned variables for each stage of the lifespan (Fig 3B). We also tried to model the association with and without considering stages. So we conducted a model selection process using the Likelihood-Ratio test to compare the quality of the fitting by various degrees. In case of ignoring stages, we found that quadratic function of y = -9343715x^2^ + 7043818x - 139328 is the best fitted ones to the relation of negative link density and number of frustrations where the Likelihood-Ratio test indicated that quadratic function was fitted to the relation better than the linear function (F-value = 401.67, p-value < 0.0001) but cubic function could not fit better than quadratic function (F-value = 1.2, p-value = 0.27). We also performed the process for the data of each lifespan stage separately and discovered that the quadratic relation remains the best-fitted model for each stage but with different fitting parameters. We reported fitted parameters and related statistics of model selection processes stagewise and totally at the S3 Table.

Since various stages have different fitting parameters, we decided to compare fitted models of different stages. So we performed an ANCOVA test to investigate the relation of negative link density and the number of frustrations with considering the lifespan stage as a covariate factor. We found a significant interaction effect between the stages and the negative link density in the prediction of the number of frustrations (F-value = 7.85, p-value = 3.33e-06). This result reveals significant differences between stagewise regressors.

### 2.3. Topological features of brain negative subnetwork

We revealed the importance of negative links in brain network balance in previous work [27]. Also, we obtained a correlation between negative link density and frustration appearance in the last section. So we decided to trace the collective behavior of brain negative links across the lifespan. In this regard, we divided brain signed network into positive subnetwork and negative subnetwork (Fig 4A) then investigate global measures of negative subnetwork over the lifespan stages as follows:

- **Degree distribution.** This measure gives us information about the network topology and the quality of link arrangement. At first, we calculated the nodal degrees of each subject’s negative subnetwork, then we extracted a degree distribution from them for each stage. It may be helpful to explain that the degree of a node is equal to the number of connected links to that node. Fig 4B indicates that all of the stages have log-normal distributions with a peak around the degree of 10. It demonstrates that major nodes have a degree near this. In addition, higher peaks belong to early adulthood and middle adulthood. This effect also describes that the brain negative network of early and middle adults respects the rule of a specific degree in a better way. We also used Maximum Likelihood Estimation (MLE) algorithm to optimize the fitting parameters of log-normal functions. S4 Table presents the best-fitted parameters and estimation error of the fitting process.
- **Global hubness**. Higher degree nodes are called hubs. Hubs are prominent components of a network because of their large number of connections. Global hubness demonstrates the multiplicity of hubs in a network. We assessed the global hubness of brain negative subnetworks using Tendency to Make Hub (TMH) [27] then we compared global hubness of lifespan stages. Kruskal-Wallis test shows significant differences between global hubness of subjects between lifespan stages (H = 34.1, d.f = 4, p-value = 7.09e-07). Fig 4C indicates the within-stage TMHs and significant between stages differences. S5 Table also reports Dunn’s post hoc statistics related to between stage comparisons. As the figure shows, the global hubness of children, adolescents, and early adults is significantly greater than middle and late adults.
- **Global efficiency.** Path length of two connected nodes is defined as the number of nodes located between them. Every two nodes may have some paths, the smallest one is called the shortest path length. The average of all of the shortest path lengths represents the easiness of information transmission in a network where networks with lower average shortest path length are more efficient and transmit information between nodes easily [33]. Kruskal-Wallis test indicates significant differences between global efficiency of lifespan stages (H = 32.35, d.f = 4, p-value = 1.62e-06). Fig 4D demonstrates that negative subnetwork of children and adolescents have lower path length and higher efficiency compared to early and middle adults. S6 Table also represents statistics related to the between stage differences obtained from Dunn’s test.
- **Global clustering.** A cluster is tightly connected nodes in the network and matters for functional segregation. The global clustering coefficient indicates the tendency of a network in cluster formation [33]. We evaluated the ratio of triangles to triplets of negative subnetworks to assess their global clustering, then we compared evaluated global clustering between lifespan stages. We found significant differences between the clustering coefficient of stages using the Kruskal-Wallis test (H = 53.59, d.f = 4, p-value = 6.4e-11) and Dunn’s post hoc tests (S7 Table). Fig 4E shows that childhood and adolescence stages have significantly greater global clustering compared to adulthood.

### 2.4. Sex differences in requirement to change

**Fig 5:**
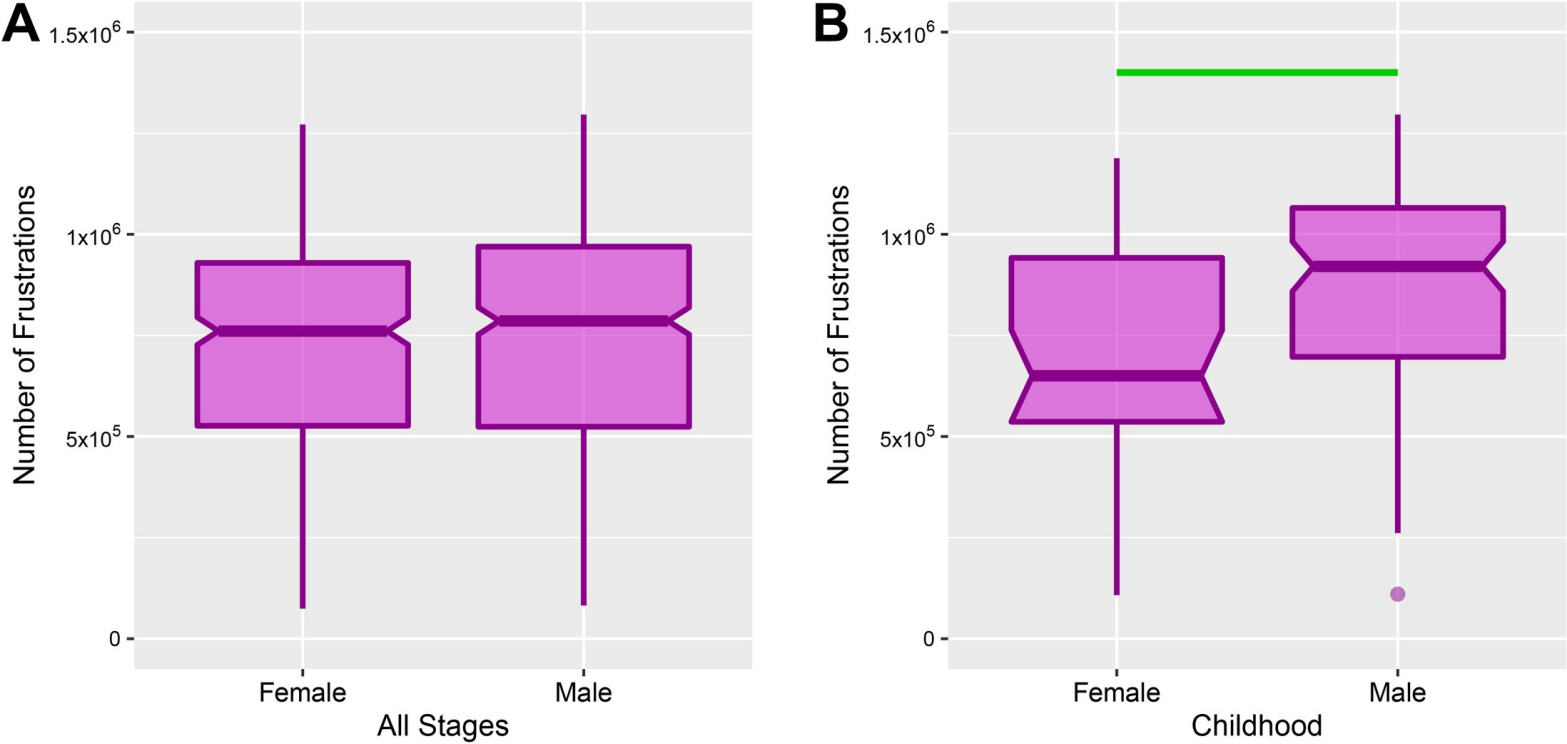
Gender differences in requirement to change of functional brain network. Comparisons of the number of frustrations between male and female subjects of all subjects disregarding lifespan stages **(A)** and children **(B)**. The notched regions of boxes indicate median and 95% confidence interval of the median and green horizontal line denotes between gender significant difference for childhood stage (p-value **≤** 0.05).

We also investigated sex differences in requirement to change of functional brain network. Because of the non-normality of distributions, we used the non-parametric Mann-Whitney test to compare the number of frustrations between male and female subjects. We could not find any significant sex-dependent differences when we ignored stages (U-value = 72684, p-value = 0.13) (Fig 5A). However, we discovered a significant difference in the requirement to change of children where boys brain networks have more number of frustrations than girls brain networks (u-value = 949, p-value = 5.29e-03) (Fig 5B).

## 3. Discussion

The brain is a learning machine that is continuously altered in exposure to new situations. Neuroplasticity is the brain’s ability to modify changes. But what requirement convinces the brain to use neuroplasticity? We studied this question in the brain network and in the level of functional connections organization. We assessed the requirement to change of the functional brain network by counting available frustrations. Frustration is a systematic dissonance between elements that persuade the system to change.

In this research, we compared the number of brain network frustrations over the lifespan stages and found a u-shape functionality, where the highest level of requirement belongs to the early and late stages of life and the lowest demand occurs in early adulthood. Then we focused on brain’s negative subnetwork because of the important role of negative links on frustration formation. We discovered a strong correlation between network size (negative link density) and the number of appeared frustration. We found that the negative brain sub-network has a special topology by log-normal degree distribution with a specific peak. We also observed that global topological features of hubness, efficiency, and clustering alter by adulthood.

### 3.1. Association with neuroplasticity

**Fig 6:**
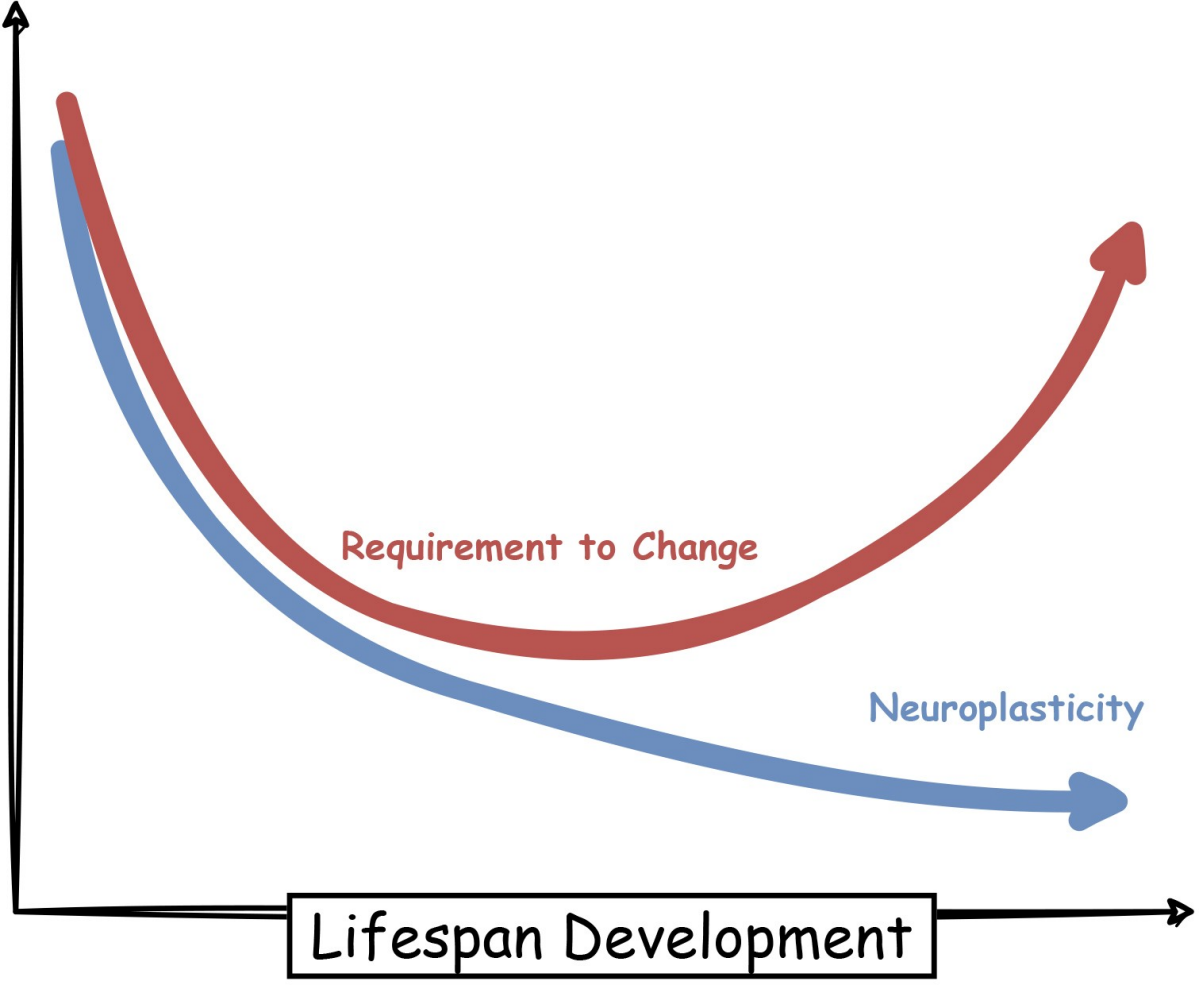
Schematic describing the alteration of neuroplasticity and requirement to change over the lifespan.

Lots of research are focused on the study of neural changes across the lifespan and many studies reveal mechanisms behind early-stage neural development and late-stage neural degeneration [16, 19, 34–36]. We know neuroplasticity is a well-known concept of the field and is defined as the ability of neural system modification [8, 10] and many studies emphasized the decline of neuroplasticity during the lifespan [37, 38]. In this research, we introduced a new attribute of the requirement to change and claim that simultaneous study of these two features gives more information to us. So we decided to appose them and simultaneously consider decreasing functionality of the neuroplasticity and u-shaped functionality of the requirement to change (Fig 6). In this way, we can infer that highly plastic brain responds to great changing requirements in the early stages and develops brain functionality as well; the brain matures in adulthood when it has the lowest requirement to change and neuroplasticity is not very useful; while lower plasticity of the elder brain can not satisfy its great requirements that may root in the wild structural deformations, so the brain can not successfully manage their functions and this weakness causes a decline in cognitive abilities of late stages.

### 3.2. Association with cognitive abilities

Behavioral studies emphasize that cognitive abilities have mainly an inverted u-shaped relation with age [35, 39, 40]. They point out cognitive functions such as memory, attention, and inhibitory control grow from childhood to early adulthood then reduce in older age. Contradictory, we showed that the requirement to change of functional brain network alters in a u-shape way over the lifespan. Our results may propose a negative association between frustration and cognitive performance that needs further investigation.

We found that early adults have the smallest number of frustrations, more structural balanced, and the weakest requirement. It corresponds to Wang et.al. studies on the segregation-integration balance. They indicated that balance between segregation and integration of brain network in young adults supports diverse cognitive abilities and the balance is correlated to better memory [41–43].

### 3.3. Topological correspondence

In our previous work [27], we had fixed negative link density and studied the effect of the negative link topology on the brain network balance. In this research, we studied topological features of brain negative subnetworks and their relations with frustration in a case of inconsistency of negative link density and where network frustration and negative link density are correlated. We understood that the brain negative subnetwork keeps its topology type over the lifespan, although its global topological features alter by adulthood (Fig 4). S6 Fig also represents correlations of the number of frustrations with both efficiency and clustering, although we think it’s a consequence of correlation of negative links density with them since they are intensive properties and depend on link density. In addition, we had found an exponential degree distribution for negative brain network in previous work [27], here, we modified the last result by decreasing the size of bins and discovered a log-normal behavior with a peak around the degree of 10.

### 3.4. Limitations and considerations

We collected multi-site neuroimages with various values of scan parameters and we only fixed functional repetition time (TR = 2s). Moreover, lifespan stages had different numbers of subjects which might threaten the validity of the multistage comparisons, but it was not problematic since our dependent metrics were distributed non-normally and we used the Kruskal-Wallis test that is feasible to unequal sample size. It should be mentioned that we did not carry out global signal regression on BOLD signals. Consequently, because of the low amount of negative links and dissimilar importance of positive links and negative links, we ignored any thresholds on functional connections and considered the signed networks fully connected. However, we compared the number of frustrations over the lifespan stage after a thresholding process in S4 Fig. To check the reliability of the requirement to change we also used brain regional activations obtained based on the AAL atlas then investigated network frustration over the lifespan (S5 Fig).

### 3.5. Conclusion and future directions

In this research, we highlighted attribute of requirement to change and introduced a network-based metric to assess it. Then we concluded that this attribute alters over the lifespan in a u-shape type functionality and discussed its relation to neuroplasticity and cognitive performance. Furthermore, we propose the study of the requirement to change besides other neural changing attributes.

Brain negative subnetworks have an effective role in the balance of network and management of between link conflicts. According to our analysis, we concluded that they have the same topology type with a log-normal degree distribution in all life stages, however, their global features contain hubness, efficiency, and clustering change by adulthood. These results underscore the study of brain negative connections and their collective behaviors.

Although we investigated the requirement to change of functional brain network of healthy subjects, studying this attribute in disorder subjects might be favorable. For instance, tracing the requirement to change of elders’ brain networks beside neuroplasticity might be helpful for the interpretation of neurodegenerative processes. Moreover, the requirement to change of functional brain network can be measured during cognitive tasks or from dynamic connectivity.

## 4. Materials and methods

### 4.1. Neuroimaging data

We used publicly available magnetic resonance images of ABIDE [28] and Southwest [29] datasets. These datasets include a T1-weighted structural MRI and a resting-state fMRI scan in the same session for each participant. We chose early and late stages subjects from ABIDE and Southwest, respectively. In this regard, we selected all healthy subjects whose resting-state images were collected in eyes open condition with a functional repetition time of 2 seconds. We selected images with the same TRs because various time points and different recording times may affect the conformity of temporal correlations and functional networks. We reported the measures of imaging protocols for each study in S8 Table. After preprocessing, we excluded subjects with destructive imaging artifacts and obtained 793 subjects for further analysis. Then we divided them into five developmental stages according to Erikson’s life stages including childhood(age: 6–12), adolescence(age: 12–18), early adulthood(age: 18–40), middle adulthood(age: 40–65) and late adulthood(age: greater than 65) [30]. Table 1 demonstrates the demographics of the selected subjects. We should mention that the neuroimaging procedures were carried out in compliance with the declaration of Helsinki. All adult subjects and parents (legal guardians) of subjects under the age of 18 provided informed consent before starting the procedure. Neuroimages are collected in several sites and the acquisition protocols were approved by their licensing committees including the Research Ethics Committee of the Brain Imaging Center of Southwest University, Institutional Review Boards of the New York University School of Medicine, The Institutional Review Board of San Diego State University, the Institutional Review Board of University of Michigan, Yale University Institutional Review Board, Ethics Commission of ETH Zurich, Georgetown University Institutional Review Board, Hospital of Trinity College, and The Institutional Review Board of University of Utah School of Medicine.

### 4.2. Preprocessing resting-state images

We used FMRIB Software Library v5.0 (FSL: http://www.fmrib.ox.ac.uk/fsl) [43] and Analysis of Functional NeuroImages (AFNI: http://afni.nimh.nih.gov/afni) [44] to preprocess MRI images. In the beginning, we deobliqued structural images to FSL friendly spaces then extracted brain tissues and segmented them to gray matter (GM), white matter (WM), and cerebral spinal fluid (CSF). After that, we focused on resting-state functional images. At first, we removed the first five volumes of functional images to ensure magnetization stability. Then, we performed a slice timing correction for interleaved slice acquisitions. Subsequently, for each subject, we registered volumes of 4D functional images to preprocessed 3D structural image (native space) using the least square algorithm that optimizes three translational and three rotational variables. We also interpolated the spiking outliers of voxelwise time series using a continuous transformation and normalized the activity of each voxel to the average of its temporal activities. Afterward, we performed spatial smoothing on each 3D volume using a Gaussian kernel with Full Width at Half Maximum (FWHM) equal to 5mm. Additionally, we carried out a bandpass filtering (0.01–0.09 Hz) on temporal activity patterns to separate biologically relevant information of the times series. Subsequently, we registered 3D volumes of functional images to MNI152 standard space (2×2×2 mm^3^) by optimization of twelve variables including three translational, three rotational, three scaling, and three shearing variables. We also regressed out three translational and three rotational confounds of motions as well as WM and CSF signals from the time series of the voxels. In the end, we visually checked the quality of preprocessing and excluded subjects that had a poor quality of extracted and segmented brains and with movement parameters greater than one voxel size. We should mention that this preprocessing procedure was already applied in other works [27, 45–46].

### 4.3. Signed functional networks formation

We used MATLAB software to extract regional brain activities according to parcels of Shen’s brain atlas [31]. This atlas parcellates the brain to 268 homogeneous cerebral and cerebellar regions of interest (ROI). For each volume and each ROI, we multiplied binary masks of the ROI to intensity of all voxels then took the average of outcomes as the activation of that ROI in that volume. We performed this procedure for all ROIs and all volumes of each 4D functional image to obtain 268 time series of regional activities for each subject. It should be mentioned that we collected functional images from some studies with different recording times. Since numbers of time points may affect the statistical inference of Pearson correlation, so we regarded only initial time points equal to the time points of the shortest time series.

Usually, ROIs and Pearson correlations between their temporal activities are regarded as the nodes and links of a functional network. Although we binarized values of functional links to +1 and -1 to derive the functional signed networks in this research. In other words, we determined signed links of functional signed networks based on positive or negative signs of correlation coefficients. It should be noted that, in the main body of the article, we disregarded any thresholds on the values of functional connections and considered brain networks fully connected, however, we presented multi-stage comparisons of the number of frustrations after thresholding processes in S4 Fig.

### 4.4. Frustration of functional network

Structural balance theory that is rooted in Fritz Heider’s researches [17, 18] explains the stability and balance of interpersonal relations [21]. The theory classifies triadic relations between entities of a social system to balanced and imbalanced (Fig 2A). It entitles a triadic relation “balanced” when a friend of a friend or an enemy of an enemy is a friend and “imbalanced” when a friend of a friend or an enemy of an enemy is an enemy. The theory describes that entities of an imbalanced triad are confused about their condition and try to change their links toward a satisfying balanced state. This is analogous to the geometrical frustration of physics [47]. In condensed matter physics, frustration (geometrical frustration) refers to the unusual and conflictive arrangement of spins and a spin system attempts to resolve them. In recent years, network scientists integrate these concepts by physical formalism to investigate the balance and frustration of social and biological complex networks [25–27, 48–50]. Since frustration pushes the network to resolve its conflicts, we claim that the number of frustrations may be regarded as a measure of the requirement to change of the brain signed network.

### 4.5. Tendency to Make negative Hub

The Tendency to Make Hub (TMH) is a global hubness measure that we introduced in previous work [27]. We defined *TMH* for a network as follows:

$$TMH\;=\;\frac{\sum_{i\;=\;1}^{N}D_{i}^{2}}{\sum_{i\;=\;1}^{N}D_{i}^{}}$$

where the summations are performed over the nodes and *D*_*i*_ represents the degree of an individual node. The *TMH* is valued 1 in the case of every node connected to only one node and grows by increasing nodal degrees.

### 4.6. Average shortest path length

A network path is defined as a connected route through links from a node to another node. Path length also is determined by the number of links located in the path. Two nodes may have some paths. The shortest one is called the shortest path length. If we consider shortest path lengths between all pairs of nodes, the average of their lengths is regarded as the Average Shortest Path Length (ASPL) [32]:

$$ASPL\;=\;\frac{\sum_{i\;\neq \;j}L_{ij}}{N}$$

Where *L*_*ij*_ is the length of the shortest path between node *i* and node *j* and ***N*** is the total number of connected nodes. In addition, the efficiency of a network is defined as the inverse of the average shortest path length. Low average shortest path length facilitates information transmission in the network and increases network efficiency.

### 4.7. Global clustering

A network cluster refers to the aggregation of interconnected nodes. The clustering coefficient is defined for each node or globally for the network. The clustering coefficient of each node determines the tendency of that node to contribute in clusters. The global clustering coefficient also indicates the propensity of the network to cluster formation. Transitivity is a common measure of global clustering [51]:

$$Transitivity\;=\;\frac{3\;\times \;N_{triangle}}{N_{triple}}$$

Where *N*_*triangle*_ and *N*_*triple*_ denote the number of presented triangles and triples. “*triple*” is assigned to any triadic relations and “*triangle*” is the closed version of “*triple*”.

### 4.8. Statistical analysis

Before every multi-stage comparison, we tested the normality of the distribution of calculated measures for each stage. If the Shapiro-Wilcoxon test showed significance null rejection even for one stage, we utilized non-parametric statistics and applied the Kruskal-Wallis test to perform multi-stage comparisons and Dunn’s post hoc test for between-stages comparisons. We also corrected the p-values of Dunn’s test from false positives that may happen by the multiple comparison process using the Benjamini & Hochberg algorithm [52]. Because of the non-normality of distributions of all measures we only used this statistical process for multi-stage comparisons (Figs 2, 3A and 4C–4E).

We fitted linear, quadratic, and cubic functions to the relation of negative link density and number of frustrations stagewise and without considering stages using the "lm" function of R software [53] (Fig 3B). To select the best model, we used the Likelihood-Ratio test and compared different orders of the models. Subsequently, we performed an ANCOVA test to investigate the covariate effect of the stage on the relation of negative links density and numbers of frustrations.

We used Maximum Likelihood Estimation (MLE) method to fit log-normal distributions to nodal degrees of brain negative subnetworks (Fig 4B).

We also tested the normality of numbers of frustrations for each gender stagewise and without considering lifespan stages. Because of the non-normality of distributions, we employed Mann–Whitney U test to investigate gender differences (Fig 5).

Generally, we applied the R software [53] and some of their packages [54–56] to carry out statistical analysis and produce graphical figures. We also generated brain map of Fig 1 by the use of BrainNet Viewer toolbox [57].

We also provided the matrix of calculated features and signed networks of all subjects in the supporting information section and online repository of https://github.com/majidsaberi/RequireChangeBrainNet. Everyone can replicate the result of this research and use data to develop the work.

## Supporting information

S1 Fig Procedure of the study. (JPG)

S2 Fig Histograms of frustration numbers. Figures show the density of the number of frustrations presented in the resting-state networks stagewise and for all stages. P-values of the Shapiro-Wilcoxon normality tests are denoted in figures.
(JPG)

S3 Fig Triad type specific comparisons of the number of frustrations. **(A)** Frustrated triads with 2 positive links and one negative link. **(B)** Frustrated triads with 3 negative links. Figures show stagewise comparisons, vertical lines and notches demonstrate medians and their 95% confidence intervals, and green lines denote significant pairwise comparisons with adjusted p-value lower than 0.05.
(JPG)

S4 Fig Comparing frustrations of partially connected networks. Each figure demonstrates the stagewise comparison of the number of frustrated triads after applying a threshold on absolute values of functional connections. Medians and their 95% confidence intervals are denoted by vertical lines and notches. Green lines also determine significant pairwise comparisons with adjusted p-value lower than 0.05.
(JPG)

S5 Fig Multiple stage comparisons of frustrated triads obtained based on AAL atlas. Horizontal lines of boxes indicate median and notches determine 95% confidence interval for the medians. Green lines present significant group differences after multiple comparison corrections (p-value **≤** 0.05).
(JPG)

S6 Fig Pairwise comparison of interesting variables. The paired plot shows univariate and bivariate analysis of the variables stagewise and totally. Different colors denote different stages. Abbreviations: NLD–Negative Link Density, TMH–Tendency to Make Hub, ASPL–Average Shortest Path Length, GC–Global Clustering, FRUS–Frustration.
(JPG)

S1 Table Pairwise statistics of frustration comparisons corresponding to Fig 2. Dunn’s adjusted p-values are reported in cells and their z-values are parenthesized below them. Also, highlighted cells indicate significant comparisons with p-values lower than 0.05.
(DOCX)

S2 Table Pairwise statistics of comparisons between negative link densities corresponding to Fig 3A. Dunn’s adjusted p-values are reported in cells and their z-values are parenthesized below them. Also, highlighted cells indicate significant comparisons with p-values lower than 0.05.
(DOCX)

S3 Table Model fitting and model selection for the relation between negative link density and number of frustrations (related to Fig 3B). (DOCX)

S4 Table Best Fitting parameters to log-normal distributions of negative degrees (corresponding to Fig 4B). Estimations and standard errors of estimations are reported in cells and below them.
(DOCX)

S5 Table Pairwise statistics of comparisons between Tendency to Make Hubs of lifespan stages corresponded to Fig 4C. Dunn’s adjusted p-values are reported in cells and their z-values are parenthesized below them. Highlighted cells indicate significant comparisons with corrected p-values lower than 0.05.
(DOCX)

S6 Table Pairwise statistics of comparisons between Average Shortest Path Lengths of lifespan stages corresponded to Fig 4D. Dunn’s adjusted p-values are reported in cells and their z-values are parenthesized below them. Highlighted cells indicate significant comparisons with corrected p-values lower than 0.05.
(DOCX)

S7 Table Pairwise statistics of comparisons between Global Clustering Coefficients of lifespan stages corresponded to Fig 4E. Dunn’s adjusted p-values are reported in cells and their z-values are parenthesized below them. Highlighted cells indicate significant comparisons with corrected p-values lower than 0.05.
(DOCX)

S8 Table Study-specific scan parameters. (DOCX)

S1 Data (ZIP)
