# Supplementary figures and images for: Requirement to change of functional brain network across the lifespan
Source: PLoS One. 2021 Nov 18;16(11):e0260091. doi: 10.1371/journal.pone.0260091 (PMC8601519; doi:10.1371/journal.pone.0260091)

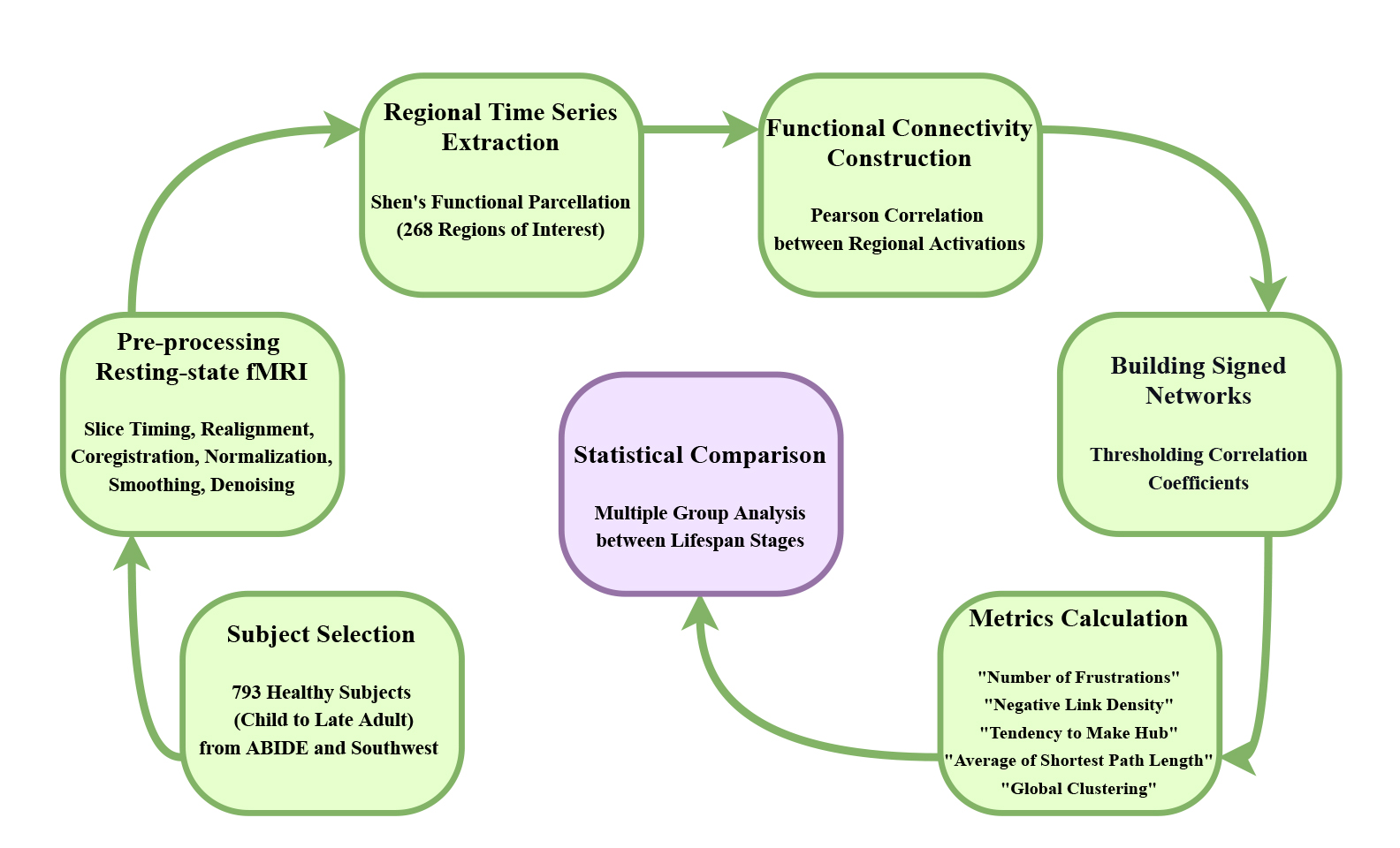

Supplement: S1 Fig — (JPG) [file pone.0260091.s001.jpg]

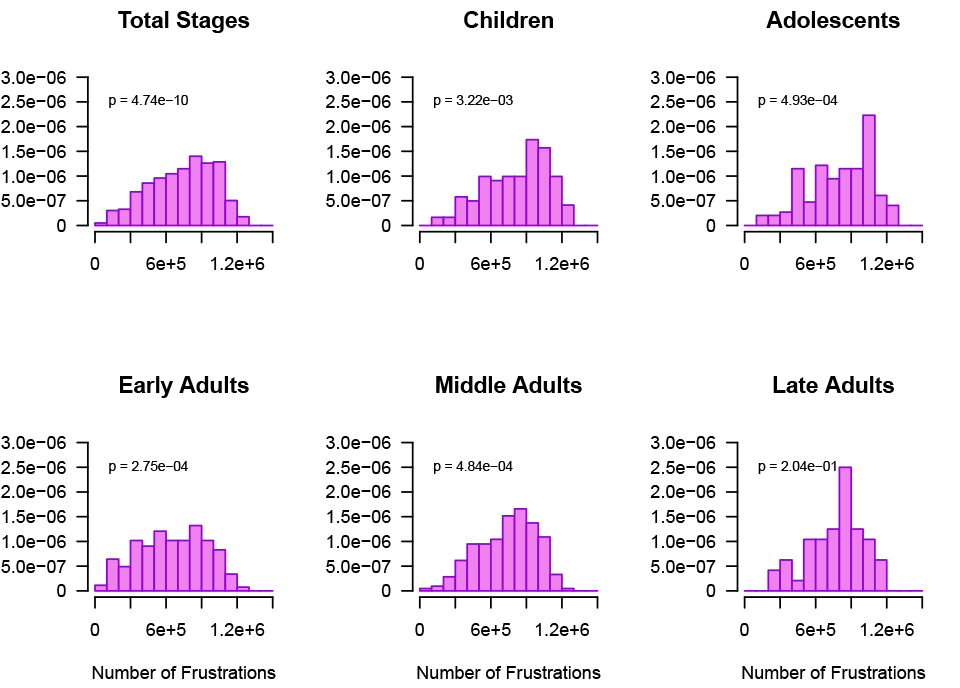

Supplement: S2 Fig — Figures show the density of the number of frustrations presented in the resting-state networks stagewise and for all stages. P-values of the Shapiro-Wilcoxon normality tests are denoted in figures. (JPG) [file pone.0260091.s002.jpg]

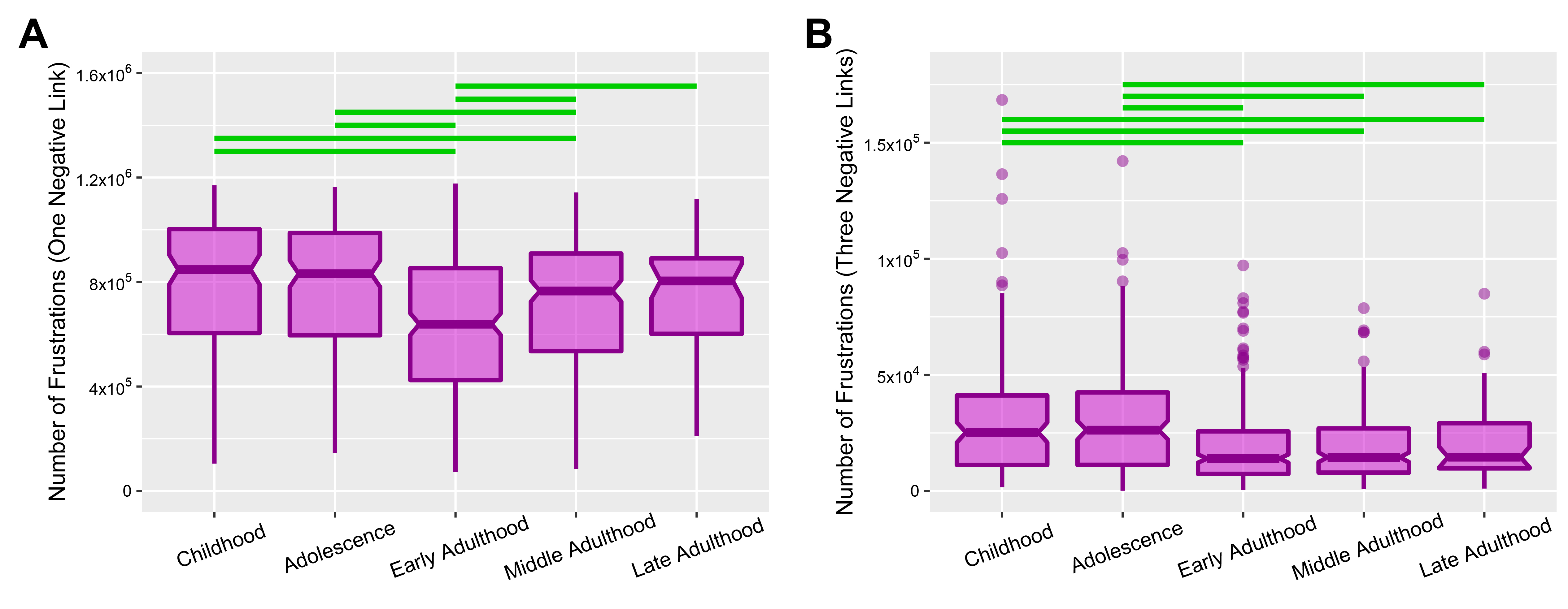

Supplement: S3 Fig — (A) Frustrated triads with 2 positive links and one negative link. (B) Frustrated triads with 3 negative links. Figures show stagewise comparisons, vertical lines and notches demonstrate medians and their 95% confidence intervals, and green lines denote significant pairwise comparisons with adjusted p-value lower than 0.05. (JPG) [file pone.0260091.s003.jpg]

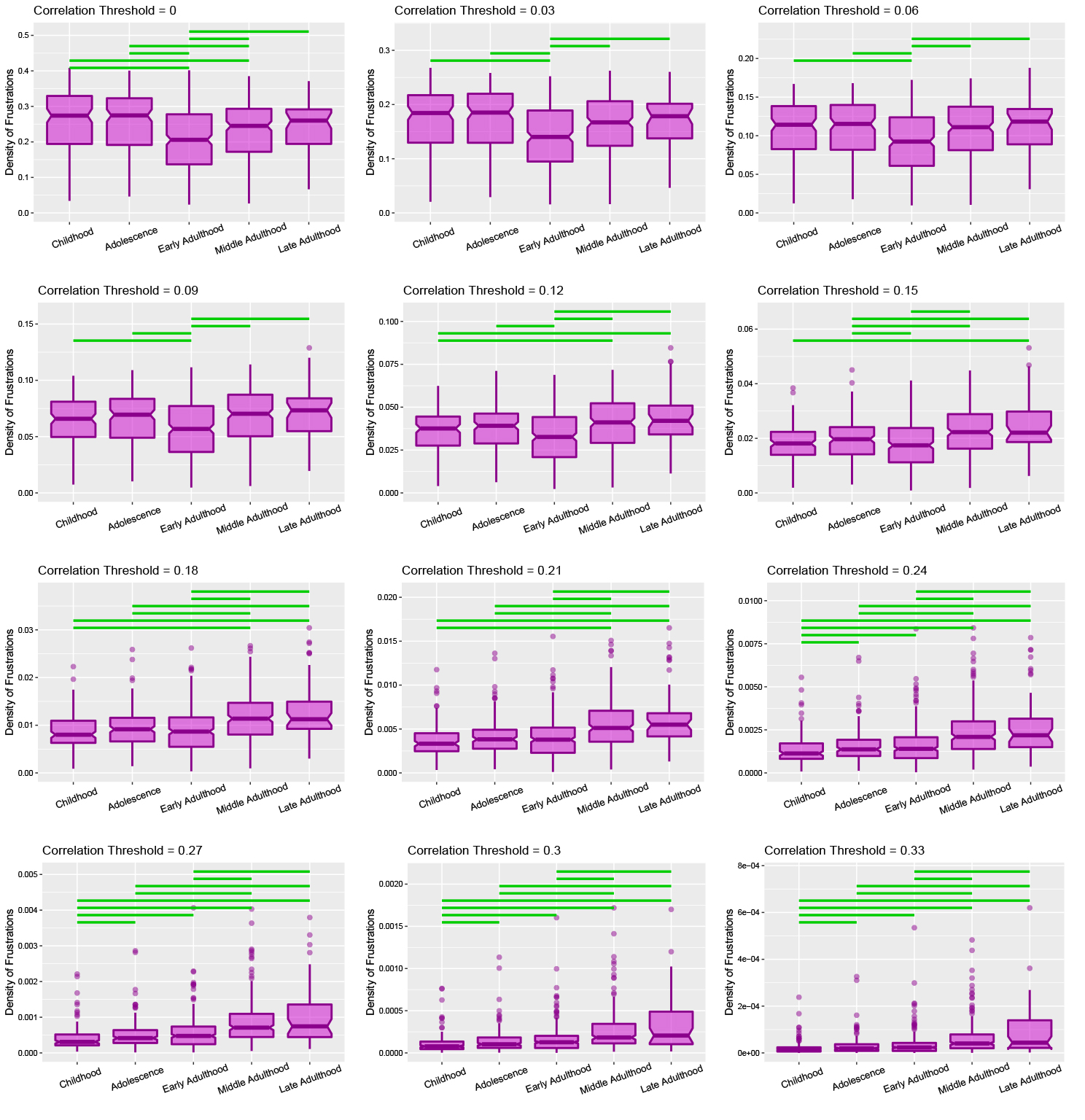

Supplement: S4 Fig — Each figure demonstrates the stagewise comparison of the number of frustrated triads after applying a threshold on absolute values of functional connections. Medians and their 95% confidence intervals are denoted by vertical lines and notches. Green lines also determine significant pairwise comparisons with adjusted p-value lower than 0.05. (JPG) [file pone.0260091.s004.jpg]

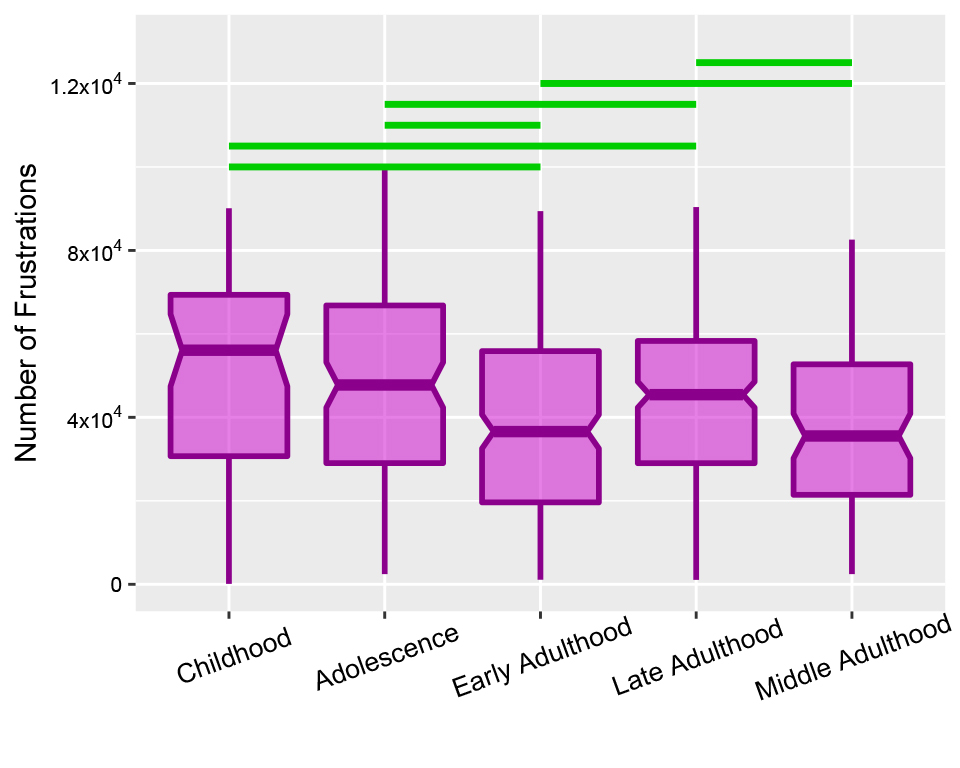

Supplement: S5 Fig — Horizontal lines of boxes indicate median and notches determine 95% confidence interval for the medians. Green lines present significant group differences after multiple comparison corrections (p-value ≤ 0.05). (JPG) [file pone.0260091.s005.jpg]

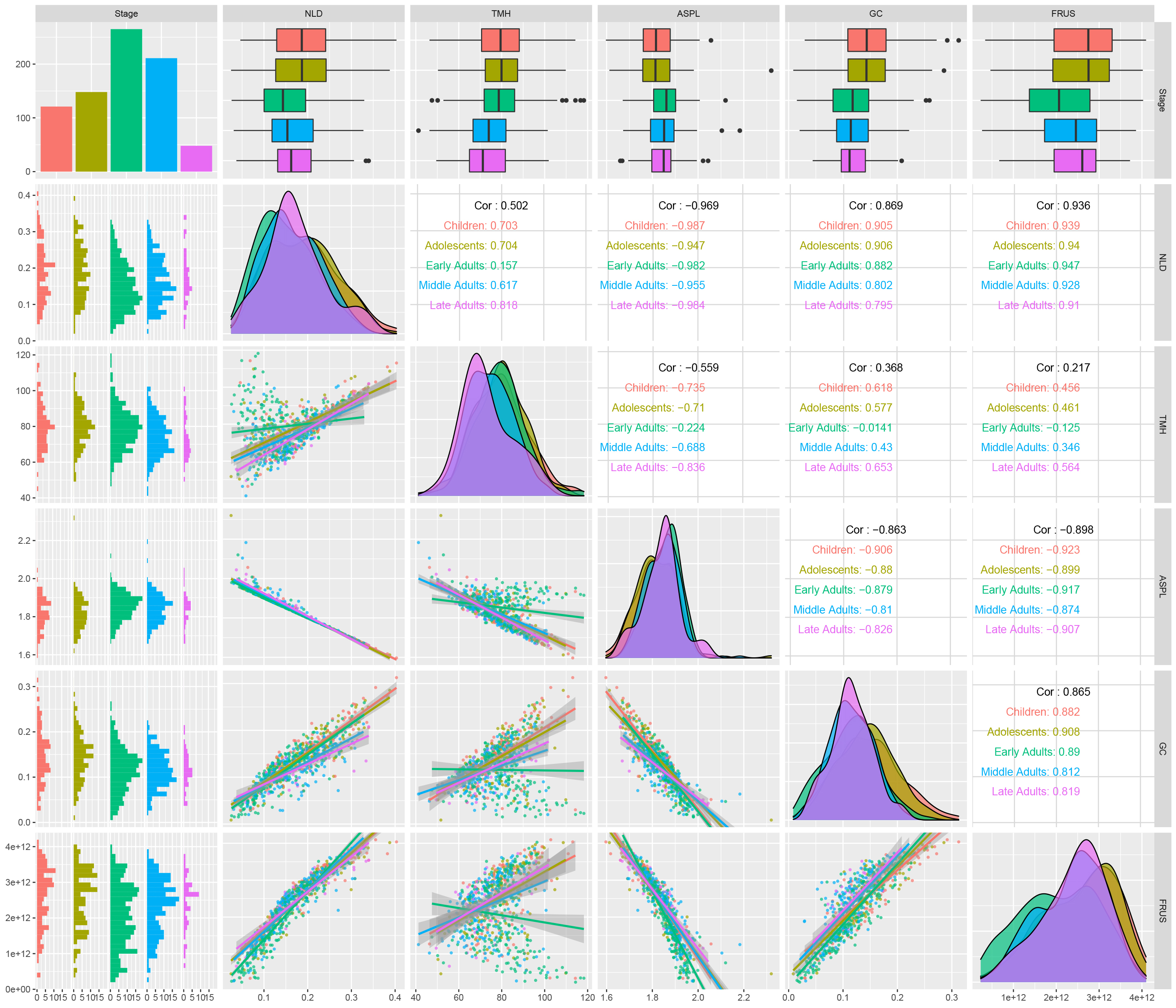

Supplement: S6 Fig — The paired plot shows univariate and bivariate analysis of the variables stagewise and totally. Different colors denote different stages. Abbreviations: NLD–Negative Link Density, TMH–Tendency to Make Hub, ASPL–Average Shortest Path Length, GC–Global Clustering, FRUS–Frustration. (JPG) [file pone.0260091.s006.jpg]
