# Supplementary material for: Requirement to change of functional brain network across the lifespan
Source: PLoS One. 2021 Nov 18;16(11):e0260091. doi: 10.1371/journal.pone.0260091 (PMC8601519; doi:10.1371/journal.pone.0260091)
Supplement: S1 Table — Dunn’s adjusted p-values are reported in cells and their z-values are parenthesized below them. Also, highlighted cells indicate significant comparisons with p-values lower than 0.05. (DOCX) [file pone.0260091.s007.docx]

**S1 Table. Pairwise statistics of frustration comparisons corresponding to Fig 2.**Dunn's adjusted p-values are reported in cells and their z-values are parenthesized below them. Also, highlighted cells indicate significant comparisons with p-values lower than 0.05.

| **Stage** | **Childhood** | **Adolescence** | **Early Adulthood** | **Middle Adulthood** | **Late Adulthood** |
| --- | --- | --- | --- | --- | --- |
| **Childhood** | - | 7.57e-01  (0.31) | 2.99e-07  (5.42) | 1.74e-02  (2.7) | 3.27e-01  (1.2) |
| **Adolescence** | - | - | 5.78e-07  (5.43) | 2.37e-02  (2.52) | 3.29e-01  (1.01) |
| **Early Adulthood** | - | - | - | 6.23e-03  (-3.11) | 2.18e-02  (-2.48) |
| **Middle Adulthood** | - | - | - | - | 5.8e-01  (-0.64) |
| **Late Adulthood** | - | - | - | - | - |
