# Supplementary material for: Requirement to change of functional brain network across the lifespan
Source: PLoS One. 2021 Nov 18;16(11):e0260091. doi: 10.1371/journal.pone.0260091 (PMC8601519; doi:10.1371/journal.pone.0260091)
Supplement: S2 Table — Dunn’s adjusted p-values are reported in cells and their z-values are parenthesized below them. Also, highlighted cells indicate significant comparisons with p-values lower than 0.05. (DOCX) [file pone.0260091.s008.docx]

**S2 Table. Pairwise statistics of comparisons between negative link densities corresponding to Fig 3A.**Dunn's adjusted p-values are reported in cells and their z-values are parenthesized below them. Also, highlighted cells indicate significant comparisons with p-values lower than 0.05.

| **Stage** | **Childhood** | **Adolescence** | **Early Adulthood** | **Middle Adulthood** | **Late Adulthood** |
| --- | --- | --- | --- | --- | --- |
| **Childhood** | - | 9.26e-01  (-0.09) | 3.3e-05  (4.51) | 4.07e-02  (2.4) | 3.48e-01  (1.08) |
| **Adolescence** | - | - | 8.28e-06  (4.93) | 2.6e-02  (2.66) | 3.39e-01  (1.18) |
| **Early Adulthood** | - | - | - | 3.38e-02  (-2.39) | 8.08e-02  (-1.97) |
| **Middle Adulthood** | - | - | - | - | 6.42e-01  (-0.55) |
| **Late Adulthood** | - | - | - | - | - |
