# Supplementary material for: Requirement to change of functional brain network across the lifespan
Source: PLoS One. 2021 Nov 18;16(11):e0260091. doi: 10.1371/journal.pone.0260091 (PMC8601519; doi:10.1371/journal.pone.0260091)
Supplement: S3 Table — (DOCX) [file pone.0260091.s009.docx]

**S3 Table. Model fitting and model selection for the relation between negative link density and number of frustrations (related to Fig 3B).**

| **Stage** | **Quadratic Model** | | | | | **P-value of Quadratic vs Linear Model** | **P-value of Cubic vs Quadratic Model** |
| --- | --- | --- | --- | --- | --- | --- | --- |
|  | **Coefficient of x^2^**  **(p-value)** | **Coefficient of x**  **(p-value)** | **Intercept**  **(p-value)** | **Adjusted R-squared** | **P-value of Fitted Model** |  |  |
| **Childhood** | -9191496  (< 2e-16) | 6991473  (< 2e-16) | -119939  (5.72e-04) | 0.94 | < 2e-16 | < 2e-16 | 0.09 |
| **Adolescence** | -8157745  (1.53e-13) | 6554055  (< 2e-16) | -90857  (0.01) | 0.92 | < 2e-16 | 1.53e-13 | 0.97 |
| **Early Adulthood** | -8034100  (1.67e-13) | 6799756  (< 2e-16) | -156866  (1.23e-08) | 0.92 | < 2e-16 | 1.67e-13 | 0.27 |
| **Middle Adulthood** | -11542504  (< 2e-16) | 7552752  (< 2e-16) | -146472  (1.45e-05) | 0.91 | < 2e-16 | < 2e-16 | 0.5 |
| **Late Adulthood** | -9878498  (2.17e-07) | 6741541  (5.91e-17) | -53522  (0.35) | 0.9 | < 2e-16 | 2.17e-07 | 0.91 |
| **Total Stages** | -9343715  (< 2e-16) | 7043818  (< 2e-16) | -139328  (3.97e-13) | 0.92 | < 2e-16 | < 2e-16 | 0.27 |
