# Supplementary material for: Requirement to change of functional brain network across the lifespan
Source: PLoS One. 2021 Nov 18;16(11):e0260091. doi: 10.1371/journal.pone.0260091 (PMC8601519; doi:10.1371/journal.pone.0260091)
Supplement: S4 Table — Estimations and standard errors of estimations are reported in cells and below them. (DOCX) [file pone.0260091.s010.docx]

**S4 Table. Best Fitting parameters to log-normal distributions of negative degrees (corresponding to Fig 4B).**Estimations and standard errors of estimations are reported in cells and below them.

| **Stage** | **Mean log** | **SD log** | **Log likelihood** |
| --- | --- | --- | --- |
| **Childhood** | 3.6  (4.81e-03) | 0.87  (3.4e-03) | -157808 |
| **Adolescence** | 3.57  (4.35e-03) | 0.86  (3.07e-03) | -192682 |
| **Early Adulthood** | 3.27  (3.51e-03) | 0.93  (2.48e-03) | -327016 |
| **Middle Adulthood** | 3.42  (3.79e-03) | 0.9  (2.68e-03) | -266314 |
| **Late Adulthood** | 3.47  (7.98e-03) | 0.9  (5.64e-03) | -61311 |
| **Total Stages** | 3.43  (1.97e-03) | 0.9  (1.39e-03) | -1007461 |
