# Supplementary material for: Requirement to change of functional brain network across the lifespan
Source: PLoS One. 2021 Nov 18;16(11):e0260091. doi: 10.1371/journal.pone.0260091 (PMC8601519; doi:10.1371/journal.pone.0260091)
Supplement: S5 Table — Dunn’s adjusted p-values are reported in cells and their z-values are parenthesized below them. Highlighted cells indicate significant comparisons with corrected p-values lower than 0.05. (DOCX) [file pone.0260091.s011.docx]

**S5 Table. Pairwise statistics of comparisons between Tendency to Make Hubs of lifespan stages corresponded to Fig 4C.**Dunn's adjusted p-values are reported in cells and their z-values are parenthesized below them. Highlighted cells indicate significant comparisons with corrected p-values lower than 0.05.

| **Stage** | **Childhood** | **Adolescence** | **Early Adulthood** | **Middle Adulthood** | **Late Adulthood** |
| --- | --- | --- | --- | --- | --- |
| **Childhood** | - | 0.72  (-0.45) | 0.69  (0.4) | 8.33e-04  (3.66) | 3.49e-03  (3.13) |
| **Adolescence** | - | - | 0.47  (0.97) | 1.03e-04  (4.41) | 9.7e-04  (3.55) |
| **Early Adulthood** | - | - | - | 2.62e-04  (4.04) | 3e-03  (3.12) |
| **Middle Adulthood** | - | - | - | - | 0.58  (0.73) |
| **Late Adulthood** | - | - | - | - | - |
