# Supplementary material for: Requirement to change of functional brain network across the lifespan
Source: PLoS One. 2021 Nov 18;16(11):e0260091. doi: 10.1371/journal.pone.0260091 (PMC8601519; doi:10.1371/journal.pone.0260091)
Supplement: S6 Table — Dunn’s adjusted p-values are reported in cells and their z-values are parenthesized below them. Highlighted cells indicate significant comparisons with corrected p-values lower than 0.05. (DOCX) [file pone.0260091.s012.docx]

**S6 Table: Pairwise statistics of comparisons between Average Shortest Path Lengths of lifespan stages corresponded to Fig 4D.**Dunn's adjusted p-values are reported in cells and their z-values are parenthesized below them. Highlighted cells indicate significant comparisons with corrected p-values lower than 0.05.

| **Stage** | **Childhood** | **Adolescence** | **Early Adulthood** | **Middle Adulthood** | **Late Adulthood** |
| --- | --- | --- | --- | --- | --- |
| **Childhood** | - | 0.88  (0.15) | 7.95e-05  (-4.32) | 1.44e-02  (-2.76) | 0.18  (-1.54) |
| **Adolescence** | - | - | 1.63e-05  (-4.79) | 6.25e-03  (-3.11) | 0.15  (-1.7) |
| **Early Adulthood** | - | - | - | 0.17  (1.72) | 0.22  (1.34) |
| **Middle Adulthood** | - | - | - | - | 0.83  (0.32) |
| **Late Adulthood** | - | - | - | - | - |
