# Supplementary material for: Requirement to change of functional brain network across the lifespan
Source: PLoS One. 2021 Nov 18;16(11):e0260091. doi: 10.1371/journal.pone.0260091 (PMC8601519; doi:10.1371/journal.pone.0260091)
Supplement: S7 Table — Dunn’s adjusted p-values are reported in cells and their z-values are parenthesized below them. Highlighted cells indicate significant comparisons with corrected p-values lower than 0.05. (DOCX) [file pone.0260091.s013.docx]

**S7 Table. Pairwise statistics of comparisons between Global Clustering Coefficients of lifespan stages corresponded to Fig 4E.**Dunn's adjusted p-values are reported in cells and their z-values are parenthesized below them. Highlighted cells indicate significant comparisons with corrected p-values lower than 0.05.

| **Stage** | **Childhood** | **Adolescence** | **Early Adulthood** | **Middle Adulthood** | **Late Adulthood** |
| --- | --- | --- | --- | --- | --- |
| **Childhood** | - | 0.99  (0.001) | 2.38e-06  (4.96) | 2.96e-06  (4.86) | 1.85e-03  (3.26) |
| **Adolescence** | - | - | 1.17e-06  (5.3) | 1.19e-06  (5.17) | 1.63e-03  (3.35) |
| **Early Adulthood** | - | - | - | 0.99  (0.11) | 0.94  (0.08) |
| **Middle Adulthood** | - | - | - | - | 0.98  (0.01) |
| **Late Adulthood** | - | - | - | - | - |
