# Supplementary material for: Requirement to change of functional brain network across the lifespan
Source: PLoS One. 2021 Nov 18;16(11):e0260091. doi: 10.1371/journal.pone.0260091 (PMC8601519; doi:10.1371/journal.pone.0260091)
Supplement: S8 Table — (DOCX) [file pone.0260091.s014.docx]

| **Dataset** | **Study** | **Final Selected Subjects** | **Voxel Size(mm)** | **S**tructural **Flip Angle (Deg)** | **Functional Flip Angle (Deg)** | **S**tructural **Echo Time (ms)** | **Functional Echo Time (ms)** | **S**tructural **Repetition Time (ms)** | **Functional Repetition Time (ms)** |
| --- | --- | --- | --- | --- | --- | --- | --- | --- | --- |
| Southwest | Southwest University | 159 | 1.0×1.0×1.0 | 9 | 90 | 2.52 | 30 | 1900 | 2000 |
| ABIDEI | NYU Langone Medical Center | 61 | 1.3×1.0×1.3 | 7 | 90 | 3.25 | 15 | 2530 | 2000 |
|  | San Diego State University | 15 | - | 8 | 90 | min full | 30 | 600 | 2000 |
|  | University of Michigan | 46 | 3.438×3.438×3.0 | 15 | 90 | 1.8 | 30 | 500 | 2000 |
|  | University of Utah School of Medicine | 32 | 1.0×1.0×1.2 | 9 | 90 | 2.91 | 28 | 2300 | 2000 |
|  | Yale Child Study Center | 17 | 1.0×1.0×1.0 | 9 | 60 | 1.73 | 25 | 1230 | 2000 |
| ABIDEII | ETH Zürich | 16 | 0.898×0.898×0.899 | 8 | 90 | shortest | 25 | 8.4 | 2000 |
|  | Georgetown University | 25 | 1.0×1.0×1.0 | 7 | 90 | 3.5 | 30 | 2530 | 2000 |
|  | NYU Langone Medical Center | 26 | 1.3×1.0×1.3 | 7 | 90 | 3.25 | 15 | 2530 | 2000 |
|  | San Diego State University | 20 | - | 8 | 90 | min full | 30 | 600 | 2000 |
|  | Trinity Centre for Health Sciences | 15 | 0.898×0.898×0.899 | 8 | 90 | shortest | 27 | 8.4 | 2000 |
|  | University of Utah School of Medicine | 11 | 1.0×1.0×1.2 | 9 | 90 | 2.91 | 28 | 2300 | 2000 |

**S8 Table. Study-specific scan parameters.**
